# Projections of functional dependence among the late middle-aged and older population from 2018-2048 in China: a dynamic microsimulation

**DOI:** 10.1186/s41256-024-00357-y

**Published:** 2024-04-29

**Authors:** Yawen Jiang, Limin Li

**Affiliations:** School of Public Health (Shenzhen), Sun Yat-sen University, Room 533, #1 West Wing of Medical Complex, 66 Gongchang Road, Guangming District, Shenzhen, Guangdong China

**Keywords:** Microsimulation, Projection, Functional, Dependence, Aging, Trend

## Abstract

**Background:**

The population of China is aging rapidly. However, the long-term trajectories of functionally dependent late middle-aged and older Chinese people are currently absent. The present study aimed to estimate the population size and proportion of late middle-aged and older adults with difficulties and dependence on activities of daily living (ADL) and instrumental activities of daily living (IADL) in China from 2018 to 2048.

**Methods:**

We constructed a dynamic microsimulation model to project the population size and proportions of late middle-aged and older Chinese people who have difficulty and dependence in ADL and IADL from 2018-2048. The model was populated with a representative sample of the target population and allowed individual-level interaction between risk factors, diseases, and health outcomes. Analyses by socioeconomic subgroups were also conducted.

**Results:**

Almost 25% and 38% of late middle-aged and older people in China will become ADL- and IADL-dependent by 2048, respectively. Also, 17% of the target population will be severely ADL-disabled by 2048. The inequity in functional status across subgroups by sex, educational level, and urban/rural residency will become substantial.

**Conclusions:**

The numbers and percentages of China's functionally difficult and dependent late middle-aged and older population will increase by magnitudes as of the mid-21st century, the pressure of which is compounded by its disproportionate distribution across subgroups. To alleviate the overwhelming challenge, efforts to improve the functional status of the underserved subpopulation should also be iterated.

**Supplementary Information:**

The online version contains supplementary material available at 10.1186/s41256-024-00357-y.

## Background

Having the world's largest number of senior population, China faces a rapidly aging society [1]. With the increase in life expectancy and decrease in fertility rate, the population of older people in China is expected to grow faster than in most other countries [1, 2]. The burden of morbidity and disability will also climb along with the aging trend. Although comparable in aging, China’s economic development is not on par with its high-income East Asian neighbors [3]. Therefore, the resource accumulation in China to support its aging society may be even more challenging. Trajectories of the older population with disability and dependence are, for that matter, imperative for sustainability-oriented policies. In particular, the planning of human resources dedicated to nursing, the design of long-term care systems, the preparation of informal care providers, and the adoption of modern disease prevention and nursing technologies will benefit from insights into the future disability and dependence burden.

Conventional cohort-based models, such as Markov state-transition models, to predict future population health status rely heavily on static health trends of age groups or age-by-sex strata [4]. By doing so, projections fail to consider the impacts of an array of demographic and physical factors and the dynamic interactions between risk factors and health status over time [5–7]. Consequently, the simulation process does not account for heterogenous individual conditions and the deterioration of their health over time.

To circumvent such methodological challenges, dynamic microsimulations have been proposed to forecast future population health status trends [6–14]. Unlike cohort-based models, dynamic microsimulations can model and track events at the individual level, thereby enabling the effects of event history on future events and the interaction of events with other individual-level factors. Although populated with a cohort of individuals, microsimulation models take each individual as a modeling unit, thus being individual-based instead of cohort-based. Researchers have exploited dynamic microsimulations to project the dependency status among future older populations in several countries. A leading example is the Future Elderly Model (FEM), which predicts the functional level of older Americans by simulating their life courses and accruing the outcomes [10, 11, 15]. Since its debut, FEM became a popular tool for forecasting the landscapes of ADL and IADL and was adapted to other economies, including Japan, Singapore, South Korea, Mexico, and several European countries [7–9, 13, 16, 17]. Another famous example is the Population Ageing and Care Simulation (PACSim) model, devised to predict future population size with functional dependency in the United Kingdom [12].

Constructing a dynamic microsimulation model, the present study aimed to create the trajectories of the functional loss of late middle-aged and older Chinese as represented by activities of daily living (ADL) and instrumental activities of daily living (IADL) dependency. The model presented here, the China Aging and Retirement Simulation (CHARISMA) model, aimed to project the ADL and IADL dependency status from 2018-2048 by accounting for a range of socio-demographic and health factors using a representative sample of late middle-aged and older people. Most functional status trajectories in China aimed to provide insights into past trends and short-term future trends [18–20]. An additional study that did focus on predictions projected disability trends without an explicit delineation of dependence [2]. The main contribution of the present study is to provide long-term projections of ADL-and IADL-dependency among both late middle-aged and older Chinese populations, which are currently absent in the literature. In addition, our findings shed light on the differences in the functional dependence statuses across subgroups defined by socioeconomic factors.

## Methods

### Outcomes of functional status

The CHARISMA model used ADL and IADL functional statuses of the late middle-aged and older Chinese population as the outcomes. The present study deferred to the China Health and Retirement Longitudinal Study (CHARLS) questionnaire that defined ADL based on the six activities: dressing, bathing and showering, eating, getting in and out of bed, using the toilet, and controlling urination and defecation [21]. Also, IADL was defined based on the five activities of managing money, taking medications, shopping, preparing meals, and cleaning house. Although the cohort study from which our data was sourced (CHARLS) asked about a sixth IADL activity of making phone calls, this question was not asked in the baseline survey [22]. Therefore, the present study did not include the sixth IADL item. Activity level was categorized into three states. Namely, the states were 1) no difficulty for any activity; 2) for at least one activity, the individual had some difficulty but could still do; and 3) for at least one activity, the individual needed help to do or could not do. Category 2 indicated some activity impairment whereas category 3 indicated disability [21]. By the end of the simulation, the percentages of people aged 50 years and over in each year through 2048 in each category were summarized for ADL and IADL, respectively. In addition to the rates of people with functional loss, the corresponding population size was also estimated by scaling up the sample size of the cohort. In what follows, people in category 2 are referred to as some ADL (IADL) difficulty population, and people in category 3 are referred to as ADL-dependent (IADL-dependent) population. For ADL, we also projected the percentage and size of the population with at least two disabilities, which indicated severe ADL disability [21, 23].

### Model design and structure

**Fig. 1:**
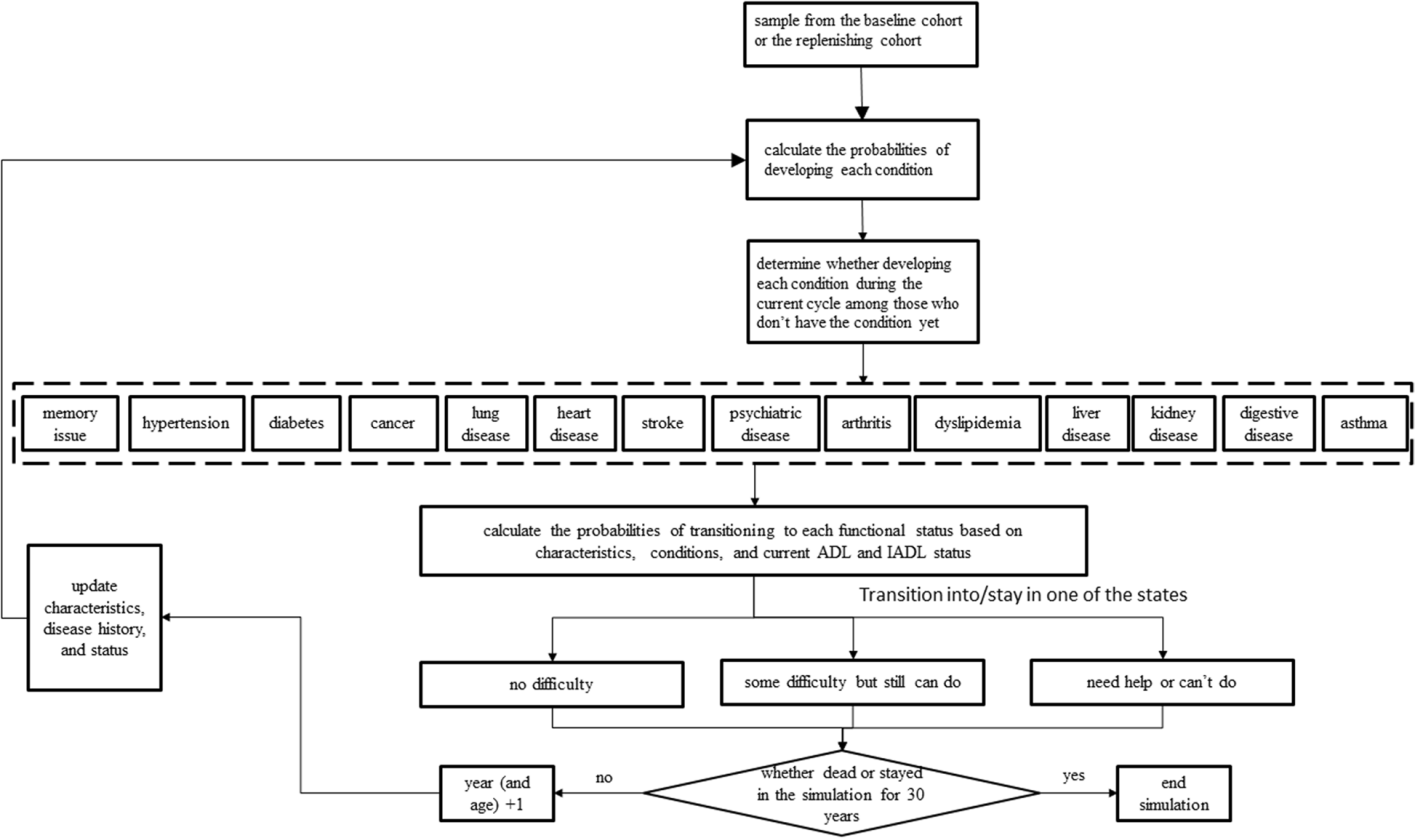
The structure of the dynamic microsimulation model

**Fig. 2:**
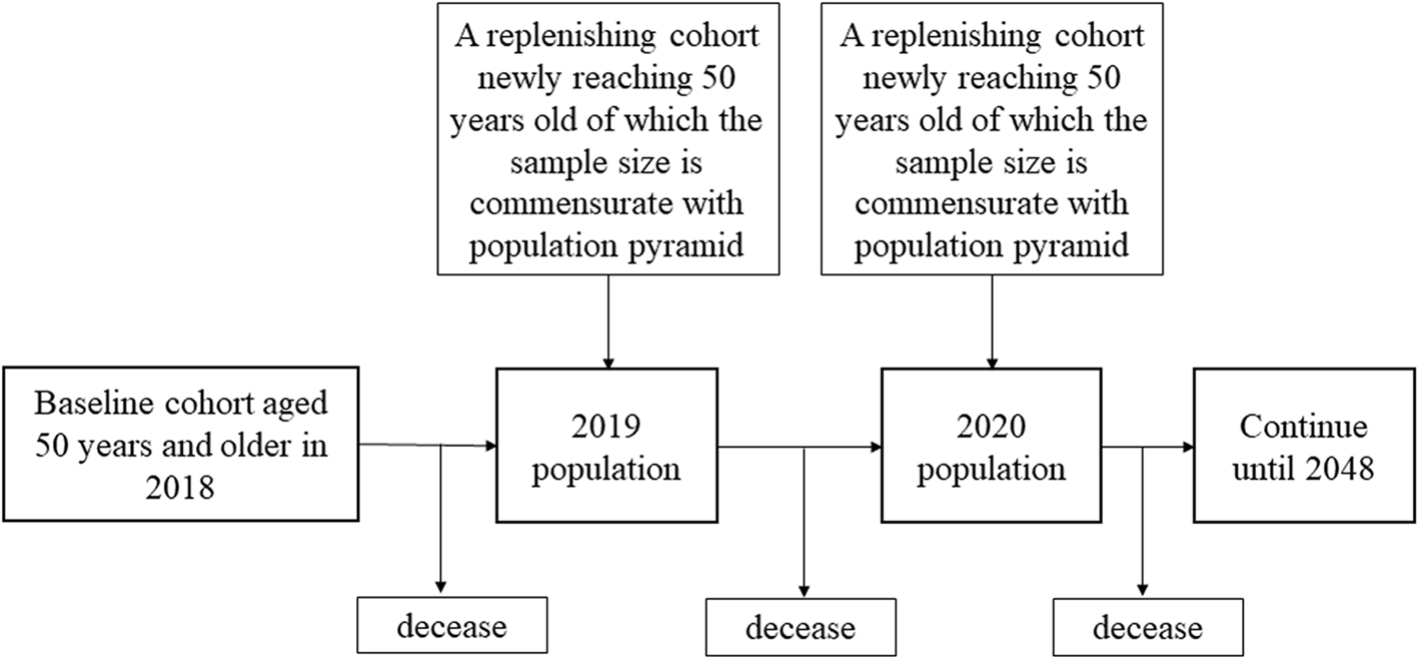
The process of simulating the baseline and replenishing cohorts

At the baseline, the CHARISMA model was populated with a sample of over 10,000 individuals representing the population aged 50 years and older in China. For every person in the model, information on socio-demographic characteristics and prevalent health conditions was used as input. Specifically, such information was used to calculate the annual probabilities of developing 14 chronic diseases if the person did not have the conditions already. On top of the simulated medical event history, the probabilities of transitioning into each of the three ADL states were calculated. The probability calculation of transitioning into each of the three IADL states followed the same approach. Against each created probability, a random variable between 0 and 1 was drawn from a uniform distribution and compared. If the random variable was greater than the probability, the development of the corresponding condition was assigned to the individual starting from the current cycle. Consistent with the nature of chronic diseases, the simulated conditions were irreversible for the rest of the life once developed [24]. The simulated status quo of health conditions was used jointly with demographic information to predict further the ordinal probabilities of ADL and IADL categories in the current cycle. The random variable technique was then used again to determine the transitioning of ADL and IADL states. Unlike chronic conditions, ADL and IADL states were not absorbing states such that individuals could move into and out of the states at each cycle. The physical conditions up to and the functional status in the current cycle subsequently entered the risk equations for the next cycle, thereby rendering a dynamic modeling process. Individuals were looped through cycles until either becoming deceased or reaching the year 2048. The structure of the model is depicted in Fig. 1. A diagram of the modeling process is shown in Fig. 2. The model was programmed using Python 3.9 and the analysis of output data was conducted using Stata 15 (StataCorp LLC, College Station, Texas, the US).

### Data sources

Individual characteristics of the simulated population and event risk equations were necessary inputs to the model. The current study primarily drew upon four waves of data from CHARLS [22]. CHARLS is a nationally representative aging survey of Chinese aged 45 years and older [22, 25, 26]. The spouses of the eligible participants were also surveyed regardless of age. The nationwide survey started in 2011, after which three follow-up surveys have already been conducted in 2013, 2015, and 2018. Data from the wave of 2018 were released in late 2020. While allowing previously non-participating family members to join follow-up surveys, CHARLS did not replenish household samples. We extracted information on age, sex, marriage status, body mass index (BMI), education, smoking, and drinking from the four waves of CHARLS data. In addition, CHARLS asked about whether the respondent has been diagnosed with fourteen types of conditions, including memory issues, hypertension, diabetes, cancer, lung disease, heart disease, stroke, psychiatric disease, arthritis, dyslipidemia, liver disease, kidney disease, digestive disease, and asthma, the information on which was also used for our analysis. We used Stata 15 (Stata Corporation, College Station, Texas, the United States) for data analysis.

### Event probability estimation

Data from the 2011-2015 waves of CHARLS were used to estimate regression equations of event risks. The reasons for using the initial three waves for probability estimation were twofold. First, it allowed extrapolation validation using 2018 observations. Second, the time step between the 2015 and 2018 waves was three years, whereas the time step was two years for the 2011-2015 waves. When estimating the event risks of the 14 chronic diseases, sociodemographic characteristics, conditions in the previous wave, and functional status in the last wave were used as predictors, all of which were relatively common predictors in microsimulation studies on population aging [8, 9, 13]. In our analysis, sociodemographic characteristics included age, sex, marriage status, BMI, education, smoking, and drinking. Age was defined as linear splines with knots at 45, 60, and 75. Similarly, BMI was defined using linear splines with a knot at 24. Also, sex was indicated using a dummy variable of male while education was grouped into below-high school, high school, and above high school. More, an indicator of whether drinking at least once a day or more often was created. Among these variables, only BMI was considered time-varying. To allow the aging effect on BMI, we regressed the latter on the linear splines of age, the indicator of sex, and the lag value of BMI. Stepwise logistic regressions with backward selection using a significance level of 0.1 for variable removal were conducted to identify the model specification for each disease’s prediction. Each logistic regression equation predicted a two-year event probability, the latter of which was then transformed into an annual probability using the declining exponential approximation of life expectancy method [27]. By contrast, ordinal logit models were used to regress ADL and IADL categories using sociodemographic characteristics, the lag values of ADL and IADL categories, and current conditions as regressors. The predicted probabilities of falling into the ordinal ADL and IADL categories were used for simulation without transformation.

CHARLS only followed up on death in 2013. Accordingly, we only relied on 2011 regressors and mortality information between 2011 and 2013 to develop the risk equation of death, the implementation of which followed that of chronic conditions.

### Baseline population

To assemble a cohort whose trajectories were to be simulated from 2018, we only sampled individuals from the 2018 wave for the baseline cohort of the CHARISMA model. To that end, we created a cohort of 13,194 people aged 50 years and older in 2018, such that each person in our sample represented 33,334 people on average if scaled up to the entire population 50 years and older in China. Further increases in the sample size might result in too many replications of the same individuals in our data source. Decreasing the sample size might result in a lack of heterogeneity and representativeness. We split the observations in 2018 with non-missing values of all required variables into age-by-sex grids, each with a pre-specified sample size based on the population pyramid [28]. Each grid was then populated by resampling from the observations in the corresponding age-sex grid of CHARLS data.

### Replenishing cohorts

To the extent that the model aimed to project the functional status of future late middle-aged and older people over a span of 30 years, it was necessary to account for the health of those newly becoming 50 years old. Therefore, a replenishing cohort of 50 years older people was introduced to the model each year. The size of the replenishing cohort was determined based on predictions of the population pyramid by age and sex in each year [28]. Since CHARLS included people aged 45 years and older, the replenishing cohorts during 2019-2023 could be sampled from those aged 45-49 years in 2018 and simulated along with the baseline cohort. However, the number of people aged 44 years and younger was limited in CHARLS and represented the spouses of the main population of interest. As such, the samples aged 44-46 years old in 2018 were re-used to create the replenishing cohort of 45 years old for each subsequent year. The sampling was not limited to those 45 years old but expanded to a 3-year bin because the sample size of people who were exactly 45 years old was relatively small. Taking 2019 as an example, a 45-year-old replenishing cohort using 2018 samples who were 44-46 years old was created based on the corresponding population size in 2019 and simulated onward, then formally became part of the simulation population in 2024 after turning 50 years old.

### Subgroup analyses

To gain insights into the future functional status of underserved populations, we also conducted subgroup analyses by sex, education level (with and without high school education), and residence area (urban and rural residents). In addition to the socioeconomic factors, we also projected the prevalence rates of ADL- and IADL-dependence for the subgroups younger than 60 years and at least 60 years old, respectively.

### Validation and sensitivity analysis

Validation is a critical step in the process of model-based simulation analysis, serving to ensure the accuracy and reliability of the model's results [29]. It involves comparing the model's predictions with observation [29]. This comparison helps to confirm that the model is functioning as expected and producing results that reflect the phenomena it is designed to simulate [29, 30]. The process of validation is typically conducted using a variety of techniques, such as regression and visualization [30]. To validate the CHARISMA model, we created another cohort using 2011 as the baseline, the process of which followed that of the 2018 cohort. The 2011 cohort was replenished and simulated onward until 2018. The equations and simulation methods remained the same as the main analysis otherwise. To conduct internal validation, we used a linear regression model to compare simulated 2013 and 2015 prevalence rates of all chronic conditions and functional status with the corresponding rates from actual CHARLS observations. R^2^ was used to quantify internal validity. An extrapolation validation was also performed, in which the simulated and observed 2018 prevalence rates of all chronic conditions and functional status were compared. The concordance between the simulated and observed outcomes for the year 2018 provides evidence to support the validity of the model, given that neither the generation of the 2011 cohort nor the formulation of the risk equations relied on 2018 data. Lacking universally agreed cutoffs of good validity, R^2^ values >0.9 were considered good fits [31, 32].

In order to address the uncertainty associated with the parameter estimates of our risk prediction equations, a probabilistic sensitivity analysis (PSA) was conducted. This technique involves the simultaneous resampling of all parameters from their respective distributions in each repetition [33]. For our study, we conducted 1,000 repetitions by resampling the parameters from the regression results distributions.

## Results

### Overall projections

**Fig. 3:**
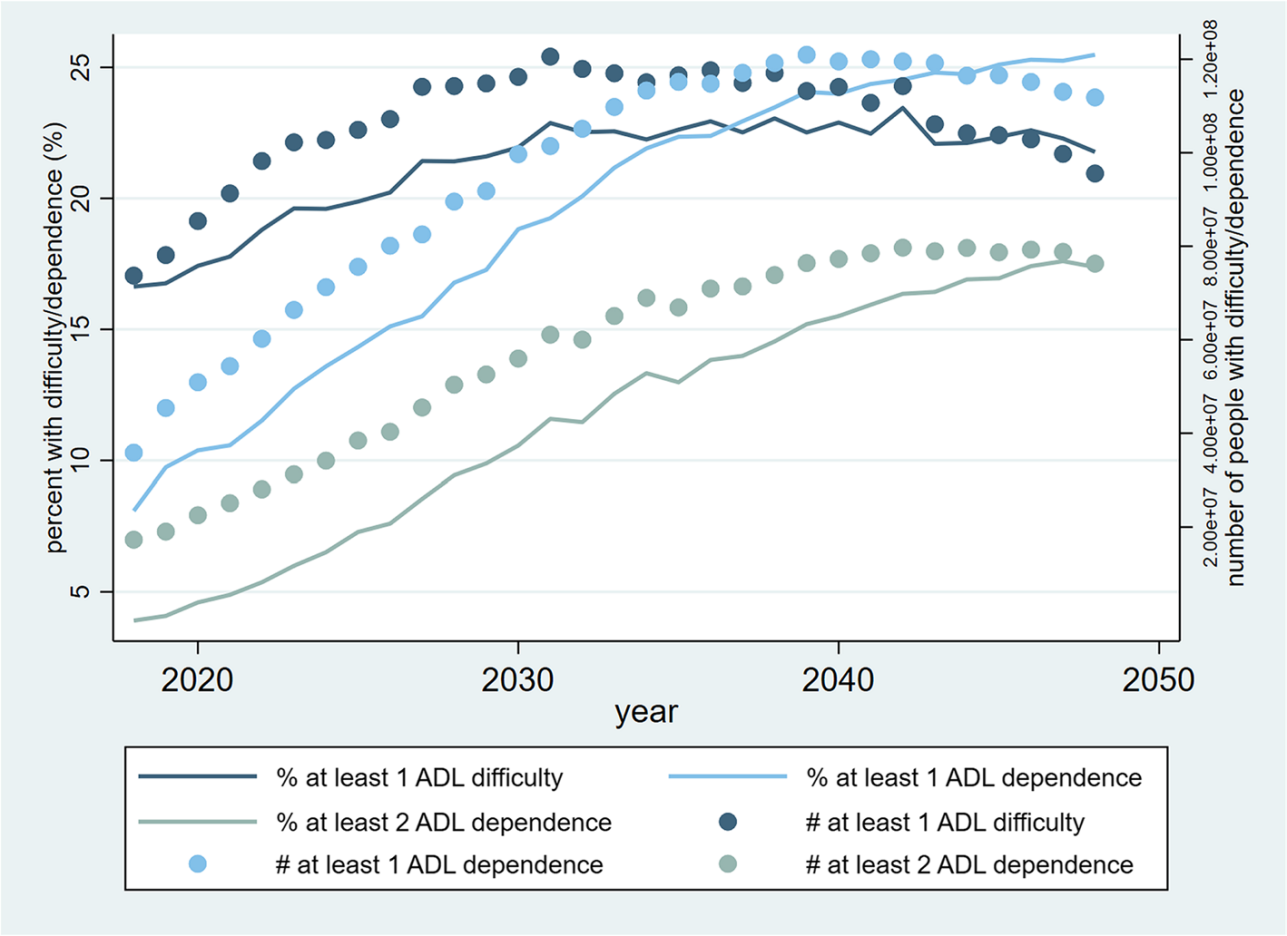
Trajectories of the percentages and population sizes of late middle-aged and older people by ADL category from 2018-2048

**Fig. 4:**
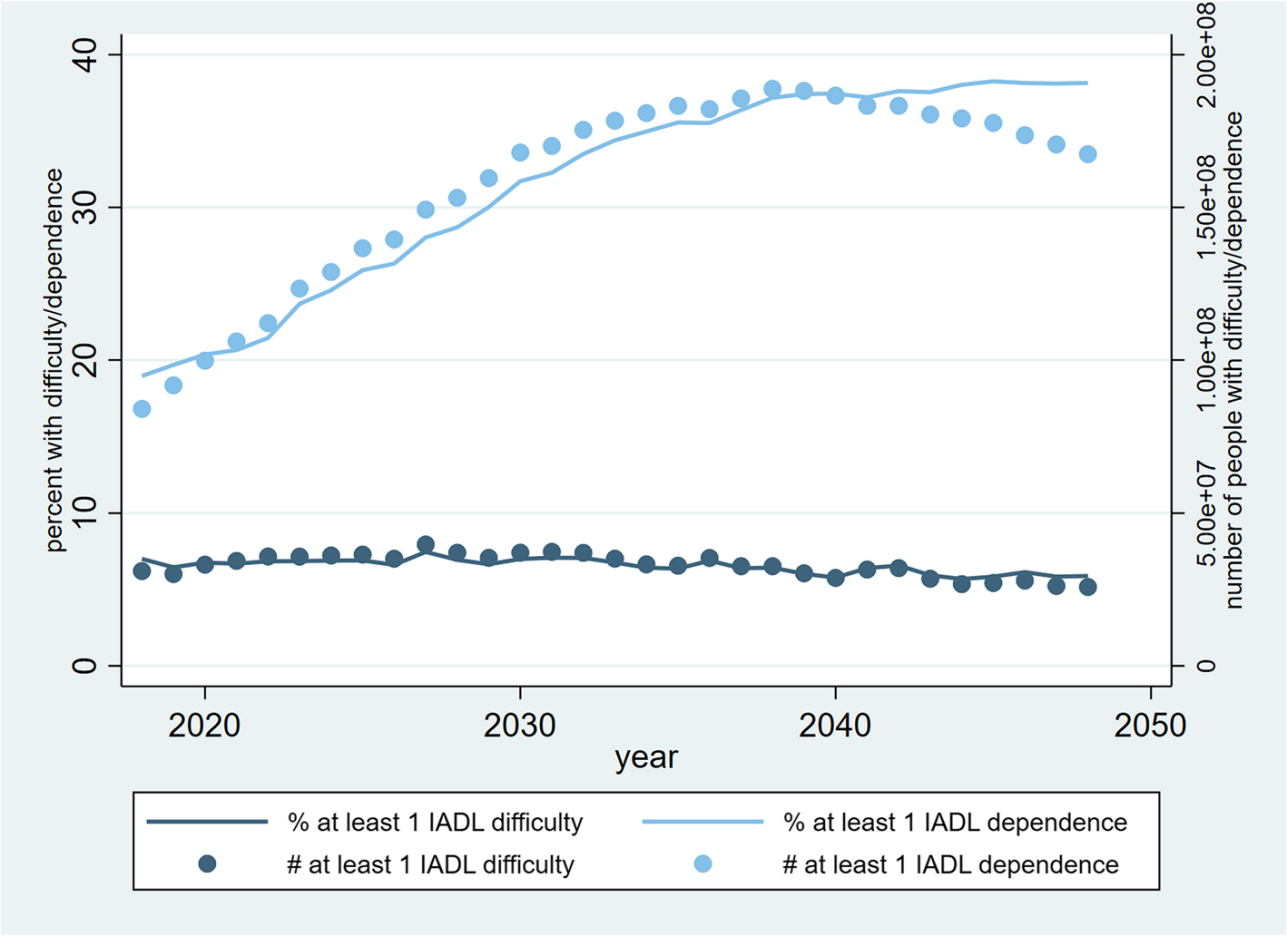
Trajectories of the percentages and population sizes of late middle-aged and older people by IADL category from 2018-2048

The study’s primary findings are presented in Figs. 3 and 4, in which Fig. 3 presents the percentages and population sizes of late middle-aged and older people in different ADL categories from 2018-2048, whereas Fig. 4 demonstrates the corresponding projections of IADL categories. The trends depicted in Fig. 3 indicate that the percentage of individuals experiencing difficulties with ADL exhibited a gradual incline from 17% in 2018 to 23% in 2031. Subsequently, the proportion plateaued and fluctuated within a narrow range, stabilizing at approximately 23% through 2048. In contrast, the percentage of ADL-dependent people soared from 8% to over 25% through 2018-2048. Similarly, the percentage of people with severe ADL disability climbed quickly from 3% to 17% in 30 years. Overall, the trends of population sizes in the three ADL categories mirrored those of percentages. Specifically, some ADL-difficulty population peaked at about 120 million in 2031 but decreased slightly to 100 million in 2048. Also, the peak of the ADL-dependent population appeared relatively late in 2039 at about 120 million. In line with the trends of ADL-difficulty and ADL-dependence, the population size of people with severe ADL disability increased from 20 million to 75 million through 2018-2048. Based on the trajectories in Fig. 4, the percentage of some IADL-difficulty people remained constant at roughly 7%, yet the percentage of IADL-dependent people nearly doubled from over 19% to almost 38%. While the number of some IADL-difficulty population remained at 30-35 million throughout the time horizon of the analysis, the IADL-dependent population skyrocketed from about 80 million in 2018 to 190 million in 2038, following which the number decreased slightly to 165 million in 2048. By summing up the numbers above, it is noteworthy that the percentage of the fully ADL-functional category substantially reduced over the years. The same was also true for the fully IADL-functional category.

### Subgroup analysis

Supplementary Figures S1-2 depict the projected trends in ADL and IADL impairment stratified by sex. At baseline and through the end of the simulation period, the proportion of women experiencing some degree of ADL impairment (both difficulty and dependence) exceeded that of men. However, the rates of severe ADL disability exhibited a comparable trajectory for both sexes, increasing from approximately 5% to 18% over the three decades. For IADL functioning, the projected percentages of both men and women experiencing difficulty remained within a narrow fluctuation range, varying by less than two percentage points over the forecast period. However, women faced substantially higher risks of developing IADL dependence during this time span compared to men, with dependence prevalence reaching 40% for women versus 30% for men by 2048. Supplementary Figures S3 and S4 illustrate the projected trends in ADL and IADL functional impairment from 2018 to 2048, stratified by educational attainment. For those with a high school education, the proportion experiencing ADL difficulty increased from 12% to 18% over the forecast period. In contrast, those without a high school diploma exhibited a proportion of ADL difficulty that was consistently higher than that of those with a high school diploma, climbing from 18% to 23%. Similarly, while the rate of ADL dependence rose from 3% to 18% among high school graduates, it spiked drastically from 10% to 28% for those without a diploma. Rates of severe ADL disability grew from 3% to 15% for high school educated individuals, which sustained lower than 5% to 18% among the less educated group. The pattern of divergent trajectories also emerged for IADL dependence, increasing from 6% to 28% among high school graduates but surging from 20% to 40% for those without high school education. Supplementary Figures S5-6 illustrate the projected trends in ADL and IADL functional impairment stratified by urban versus rural geographic residence. Over the forecast period, a marked divergence emerged in disability trajectories between these residential settings. While urban areas exhibited a gradual increase in ADL dependence from 6% to 21%, rural regions showed a more dramatic surge from 10% to 29%. Similarly, severe ADL disability prevalence climbed from 4% to 16% in urban populations, compared to a sharper incline from 5% to 20% in rural areas. This urban-rural disparity was also evident for IADL dependence, rising from 15% to 32% in urban communities but jumping precipitously from 22% to 42% among rural residents. We also explored the prevalence rates of ADL- and IADL-difficulty and dependence among the subgroups of middle-aged and older people using the cutoff age of 60 years, which was the official retirement age in China. The results are shown in Supplementary Fig S7-8. As expected, the proportions of ADL-difficulty (older: 20%-25% vs. late middle-aged: 12%-17%) and ADL-dependent (older: 10%-35% vs. late middle-aged: 5%-8%) people were greater among the older population than the late middle-aged population. The same pattern was observed among severely ADL-disabled population (older: 6%-25% vs. late middle-aged: 2%-3%). Also, the increase in the proportion of ADL-dependent people among the older population outstripped that of the late middle-aged population. In addition, although the proportion of some IADL-difficulty people was comparable across the two subpopulations (older: around 8% during the forecast period vs. late middle-aged: around 6% during the forecast period), older adults had a substantially greater proportion of IADL-dependent population (older: 28%-51% vs. late middle-aged: 9%-12%).

### Validation and sensitivity analysis

**Fig. 5:**
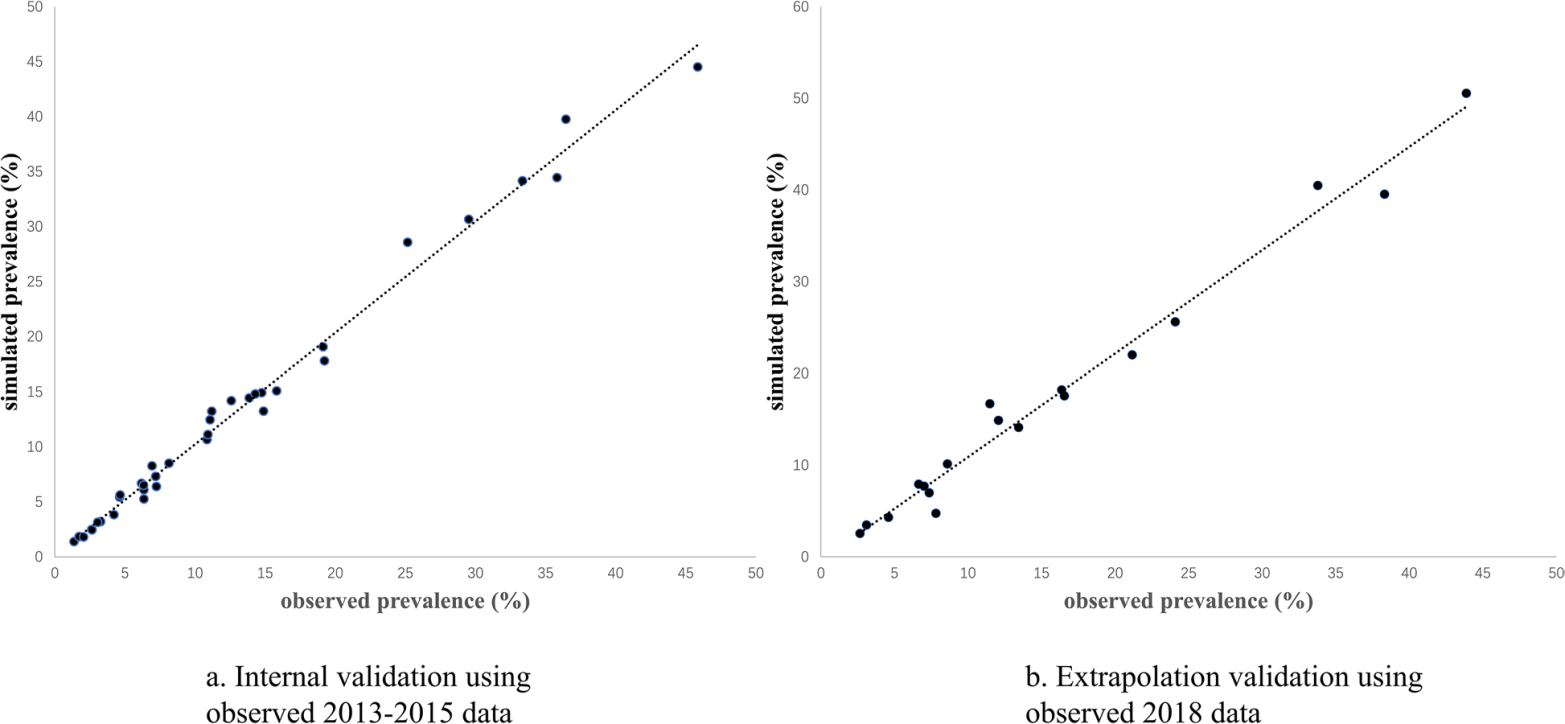
The results of internal and extrapolation validation

The internal and external validation results are plotted in Fig. 5. The R^2^ of the internal validation was greater than 0.990, whereas that of the extrapolation validation was greater than 0.982, both indicating acceptable validity. The results of PSA are presented in Supplementary Fig. S9. As expected, the uncertainty increased as time progressed.

## Discussion

In the present study, we created trajectories of ADL- and IADL-difficulty and dependent populations among late middle-aged and older Chinese over 2018-2048 using microsimulation. To the best of our knowledge, the current analysis represents the first effort to forecast the future functional impairment and dependency among both late middle-aged and older Chinese population over a span of 30 years that accounts for the dynamic aggravation of risk factors and their interplay with daily activity. It also explores a new approach to estimate the future functional burden of various diseases embodied in the microsimulation of primary interest.

The findings of our study reveal several alarming prospects for the functional status of future seniors in China, which enclose critical implications for long-term care and social policies. First, the proportions of functionally dependent older Chinese people will rise rapidly in the coming decades to a comparable level in the US and UK [12, 15]. Although not all functionally dependent people experience the same degree of disability, the current preparedness of caregiver supply in the formal and informal sectors is unlikely to match the demand for nursing care created by the substantial dependency. To alleviate the challenges of the overwhelming amount of living assistance requirements, it is mandatory to deploy programs that can prepare facilities and the labor force for a super-aged society in advance. This may include investing in caregiver training programs, improving working conditions for caregivers, and exploring innovative care delivery models, such as telecare and community-based care. Second, our study underscores the importance of preventive interventions to counteract the increasing trends of functional dependence. Our microsimulation model was largely driven by the incidence of specific diseases, many of which can be partially reduced or delayed using current medical technologies. For instance, dyslipidemia factored into the risk equations for all conditions and events, with the exception of psychiatric diseases and asthma. Therefore, enhancing population-wide management of dyslipidemia could be a key strategy in reducing future disability rates among late middle-aged and older adults. It's important to note that dyslipidemia is just one example. Similar approaches can be applied to the management of an array of other diseases, each contributing to the overall reduction in functional dependence and improving the health outcomes of the aging population. Third, it is unsettling that the gap in functional status across groups by sex, education attainment, and urban/rural residence is continuously widening. Of note, people with high school education and residents in urban areas will outperform those without high school education and residents in rural areas, respectively. However, it is unlikely that socio-economically advantaged subpopulations will become the primary labor force of caregivers. To that end, the underserved population's health status should be focused on in future planning. This could involve implementing policies to reduce health disparities, such as improving access to healthcare services in rural areas, providing health education for people with low educational attainment, and addressing gender disparities in health.

Several studies have investigated functional status among China's aging population, with some examining past trends and very few projecting short-term future trends [18–20]. According to one of the studies, it is estimated that approximately 60 million individuals aged 60 and above will experience at least one ADL or IADL difficulty by 2030. Meanwhile, our own study, based on the metric of any ADL difficulty, predicts that nearly 100 million individuals aged 50 or older will experience functional impairment by 2030. Although the figures may not be comparable, they are of a similar order of magnitude. However, our study distinguishes itself in several ways. Firstly, we extend the projection period to 2048, offering a longer-term view than previous studies. Secondly, we provide a more nuanced understanding of future functional dependence by examining health gradients across subgroups defined by education, sex, and rural/urban residence. Thirdly, our study includes not only the older adults but also the late middle-aged population, a crucial component of the labor force. Finally, we also account for the change of individual risk factors and their impacts on functional loss, providing a more comprehensive and dynamic view of the future functional dependence of the population. This comprehensive approach allows us to capture long-term trends and changes, anticipate future challenges, and offer a relatively thorough overview of the future functional dependence of the population.

The strengths of the present study are manifold. First, the CHARISMA model, following the practice of other microsimulation models of population health, allowed the dynamic progression of health status based on individual characteristics. Failing to capture such mechanisms may omit the effects of disease history, thereby underestimating the cumulative loss of functional ability. Second, the individual-level simulation allowed handy sub-group analyses. Such analyses generated imported evidence on not only the overview but also the structural issues of functional dependence. Third, the validation results suggested a relatively good model performance, which provided some confidence in the outputs.

Several limitations should be noted when interpreting the current findings. First, the sample sizes of individuals in the cohorts, particularly that of the replenishing cohorts, were modest. Consequently, the characteristics of latecomers might have not been accurately represented. Second, the types of activities compatible with common definitions were limited, hampering extensive exploration of alternative definitions of functional status. Third, the impact of each condition on functional loss and dependence-free life expectancy, which is an important issue in its own right, was not analyzed. Future research should delineate the contribution of each condition to functional loss. Moreover, a limitation arises from the inherent assumption of continuity within trend prediction, which posits that the present relationship between diseases and functioning will persist unaltered. Such an assumption overlooks the contribution of active interventions to health improvement. Additionally, the sensitivity of the model to historical data introduces another layer of complexity. Inaccuracies due to anomalies, outliers, or biases present in the data used to obtain the risk prediction equations are propagated in the microsimulation.

## Conclusions

The numbers and percentages of China's functionally difficult and dependent older population will increase by magnitudes as of the mid-21st century. The socioeconomically underserved population may have higher fractions of functionally dependent population, which exacerbates the challenge of labor supply required by long-term care and highlights the need of reducing health inequity. To neutralize the increasing trend of population functional loss, preventive measures are imperative in future endeavors.

### Supplementary Information

**Supplementary Material 1.** 

## Data Availability

Data from CHARLS is publicly available to registered researchers from the website of CHARLS (http://charls.pku.edu.cn/en/). The program code of the CHARISMA model was submitted for review and is available from the correspondent upon reasonable requests.

## Abbreviations

ADL: Activities of daily living
IADL: Instrumental activities of daily living
CHARLS: China Health and Retirement Longitudinal Study
BMI: Body mass index
FEM: Future Elderly Model
PACSim: Population Ageing and Care Simulation
CHARISMA: China Aging and Retirement Simulation
