# Supplementary material for: Projections of functional dependence among the late middle-aged and older population from 2018-2048 in China: a dynamic microsimulation
Source: Glob Health Res Policy. 2024 Apr 29;9:15. doi: 10.1186/s41256-024-00357-y (PMC11057077; doi:10.1186/s41256-024-00357-y)
Supplement: Supplementary file 1 — Supplementary Material 1. [file 41256_2024_357_MOESM1_ESM.docx]

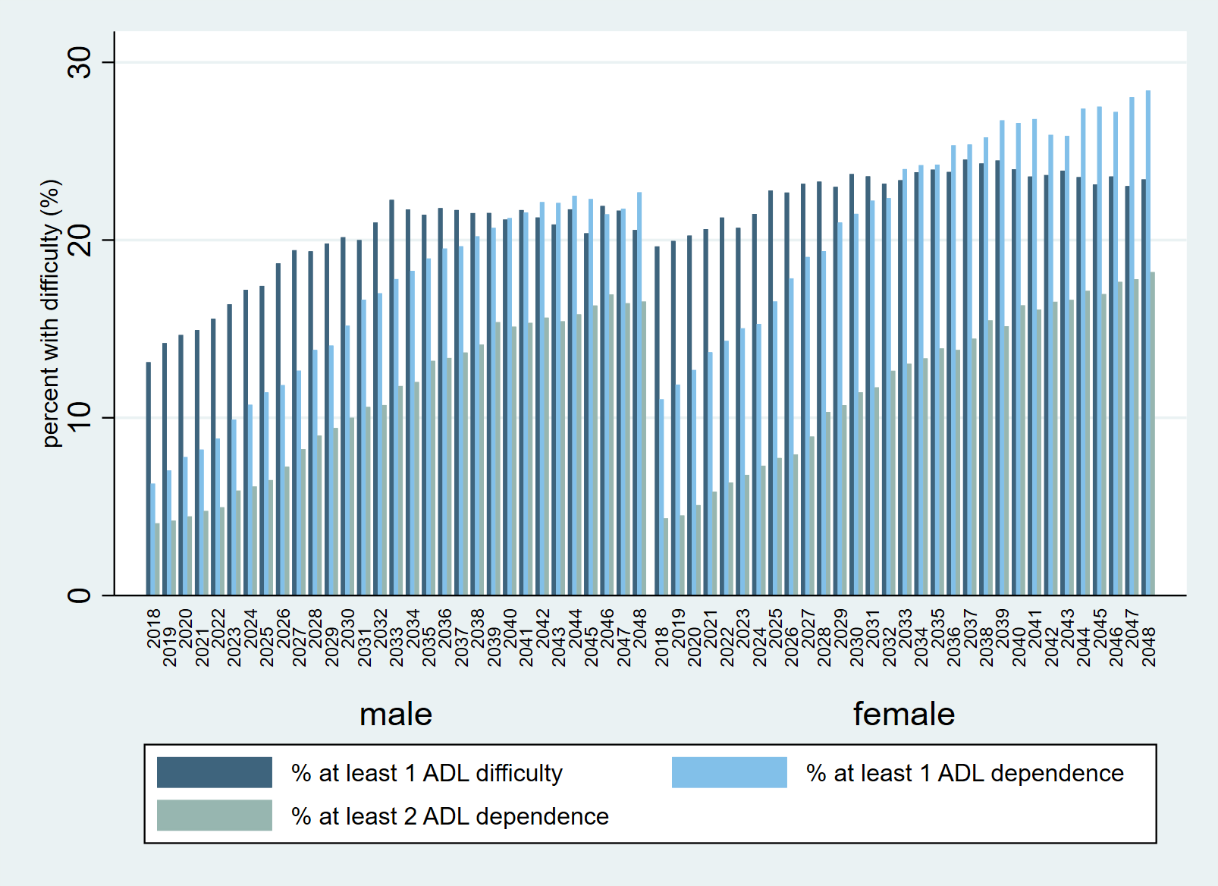


Fig S1. Projections of the activities of daily living status by sex.


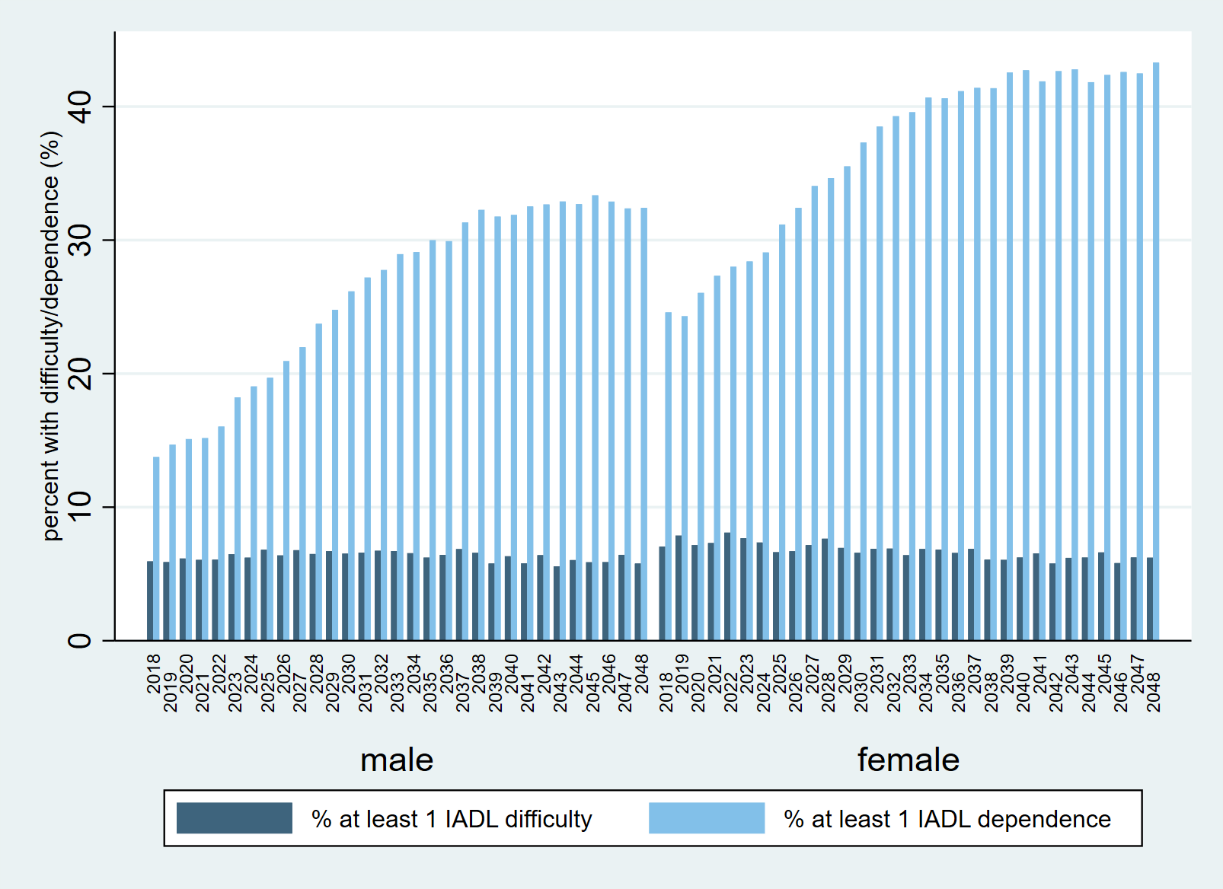


Fig S2. Projections of the instrumental activities of daily living status by sex.


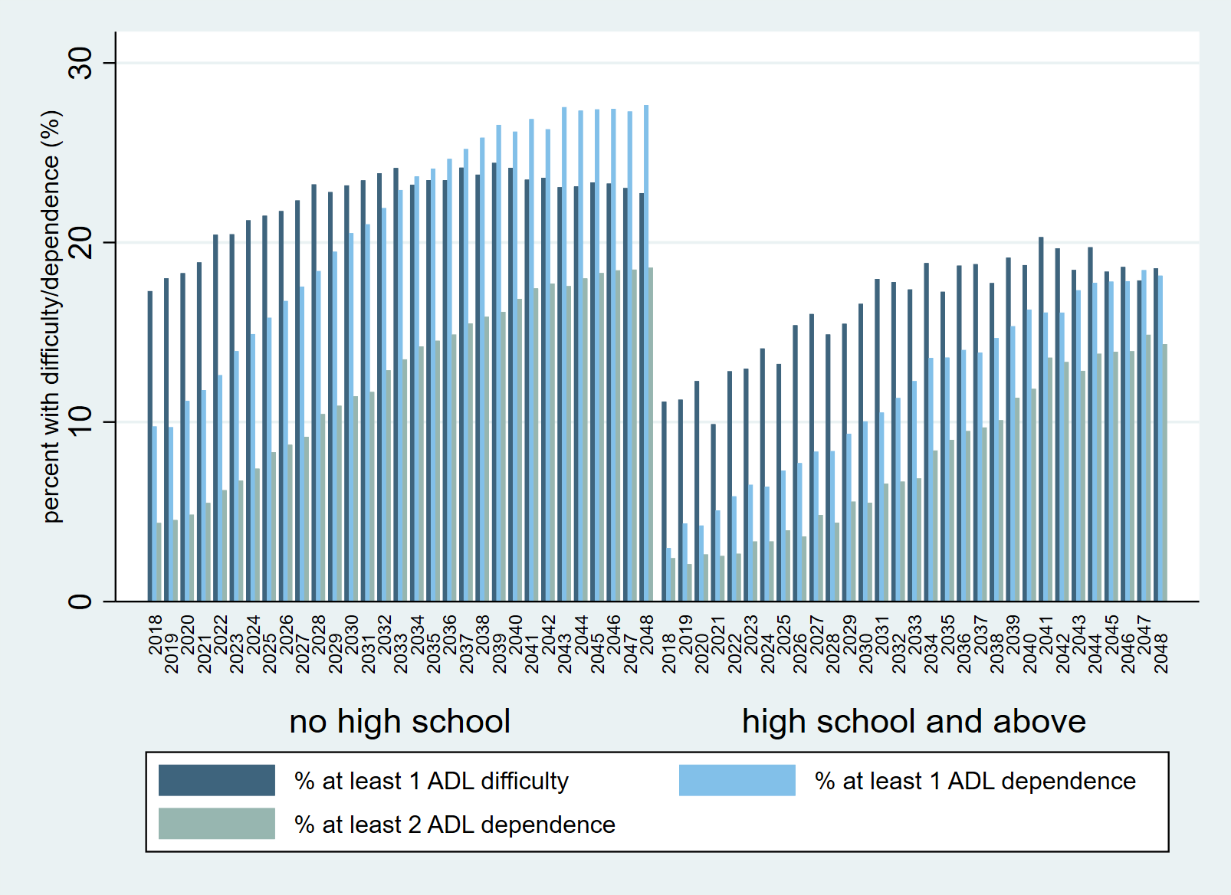


Fig S3. Projections of the activities of daily living status of late middle-aged and older people by education (high school vs. no high school).


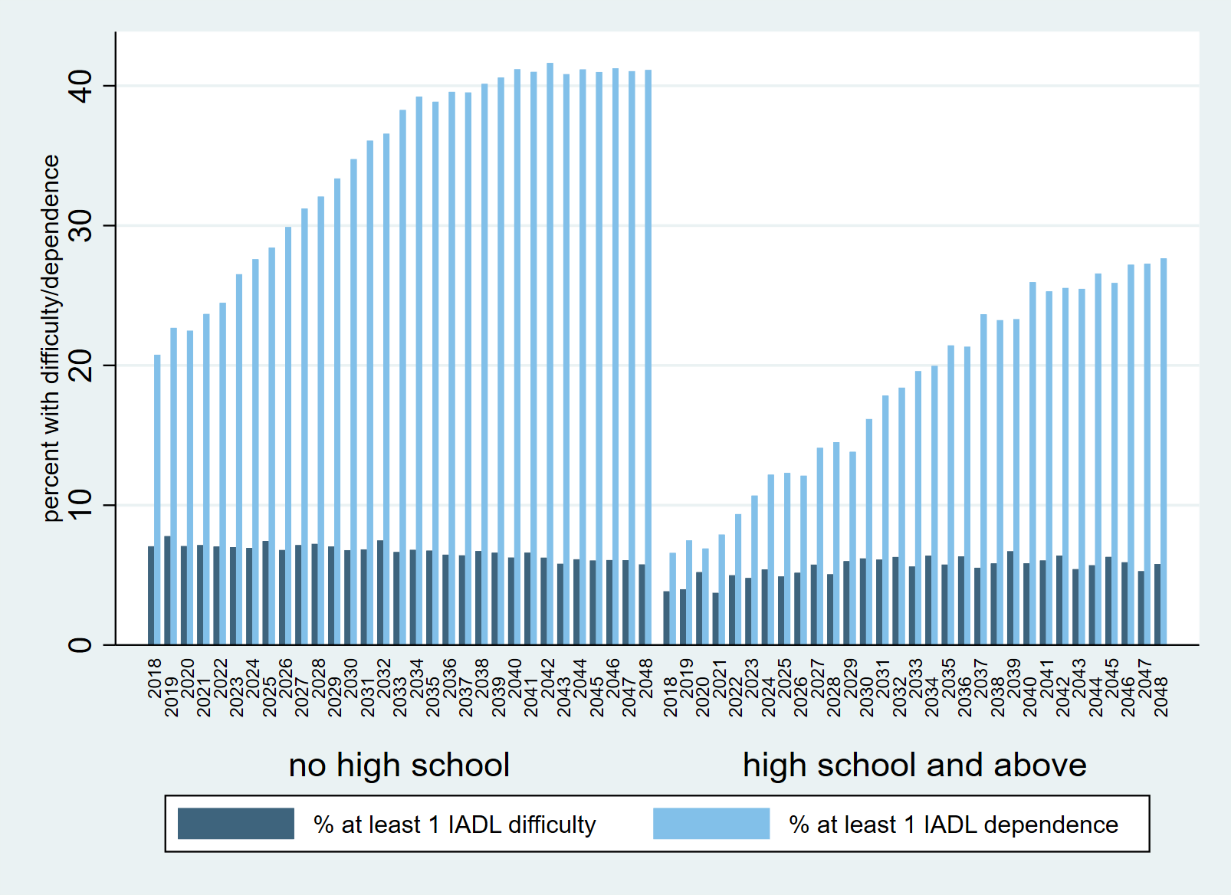


Fig S4. Projections of the instrumental activities of daily living status of late middle-aged and older people by education (high school vs. no high school).


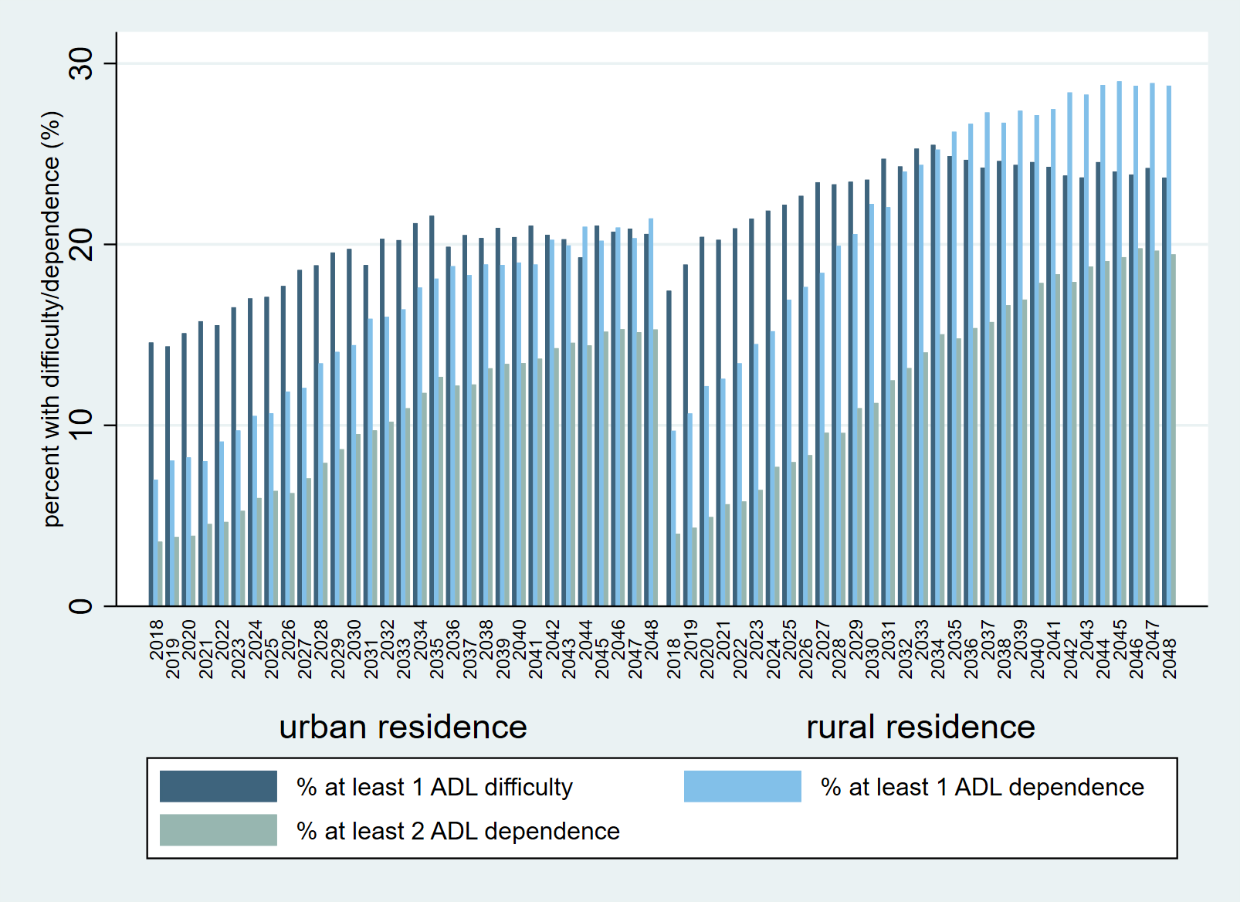


Fig S5. Projections of the activities of daily living status among late middle-aged and older urban and rural residents.


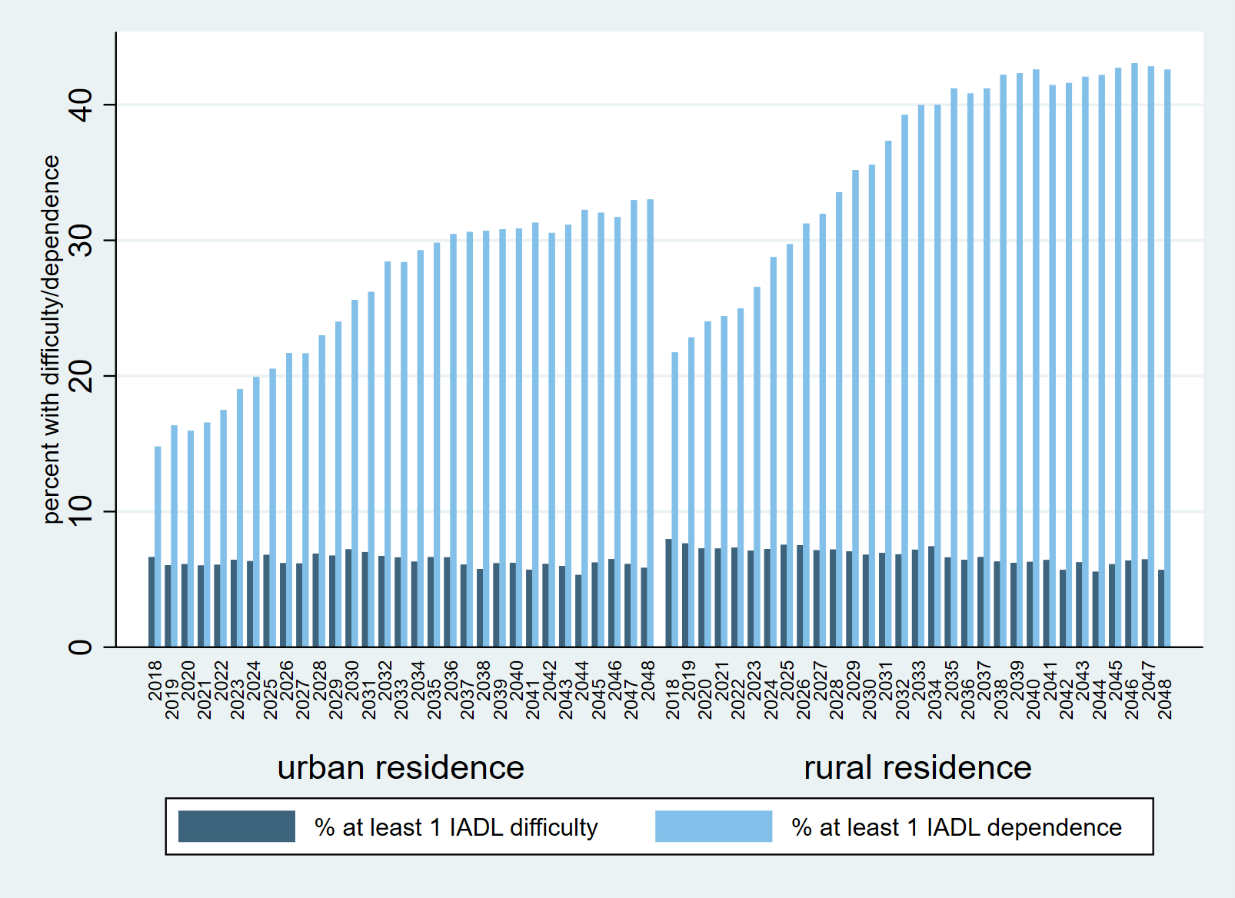


Fig S6. Projections of the instrumental activities of daily living status among late middle-aged and older urban and rural residents.


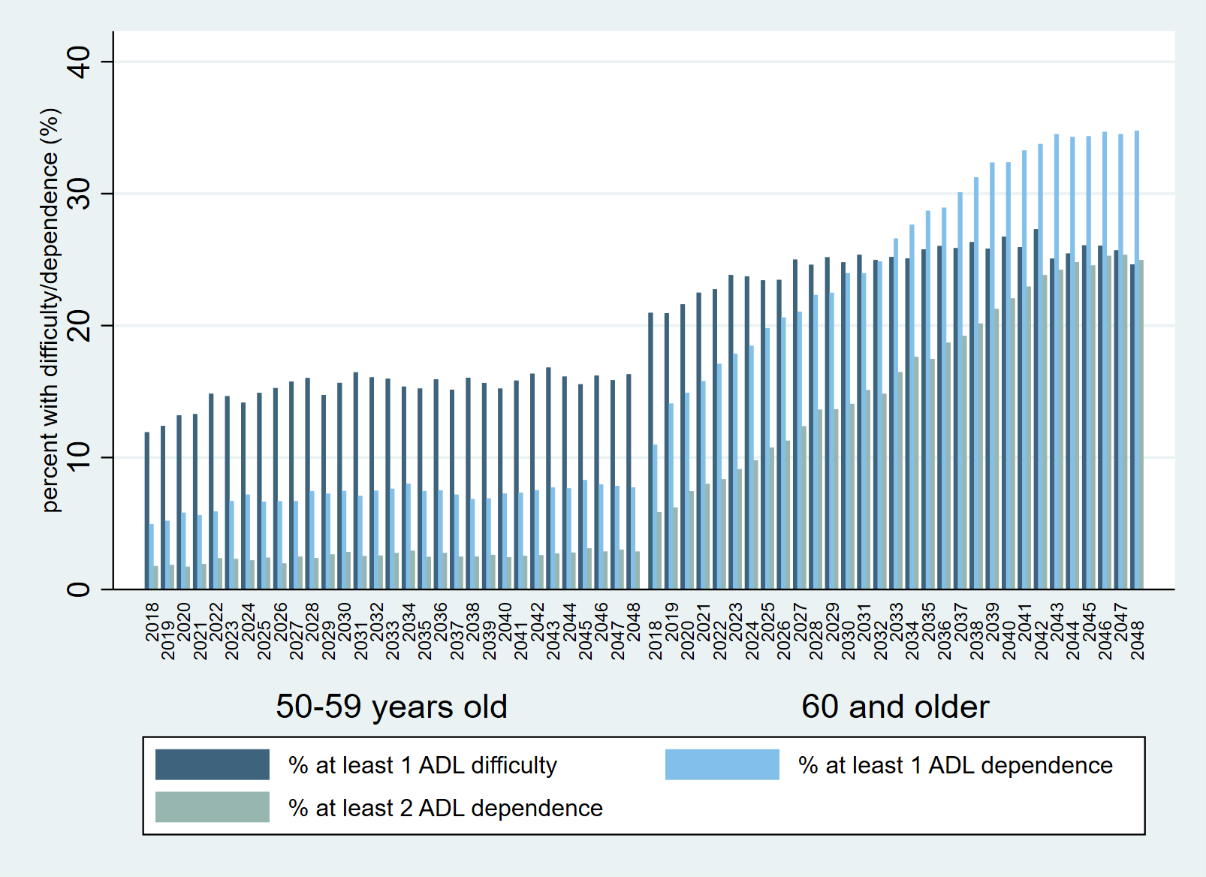


Fig S7. Projections of the activities of daily living status among the subgroups who were younger than and at least 60 years old, respectively.


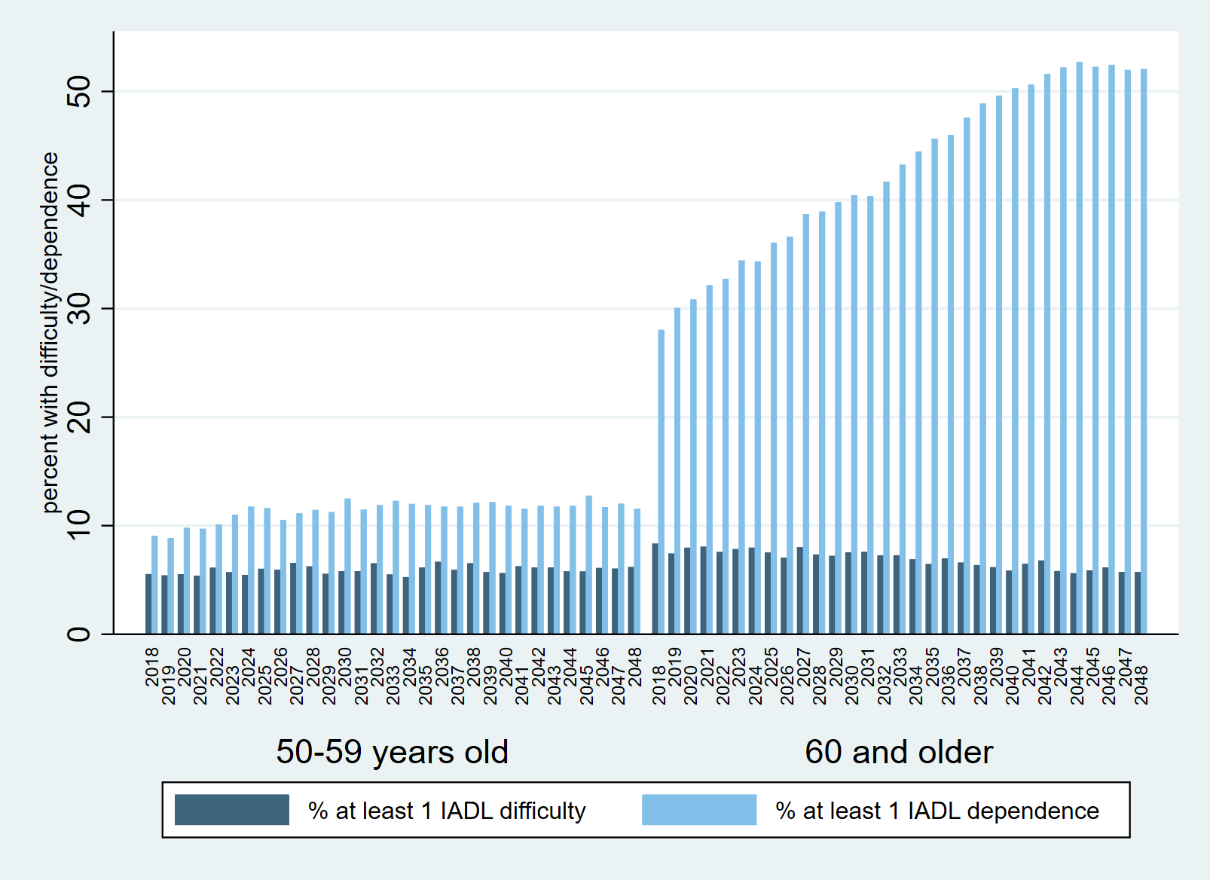


Fig S8. Projections of the instrumental activities of daily living status among the subgroups who were younger than and at least 60 years old, respectively.


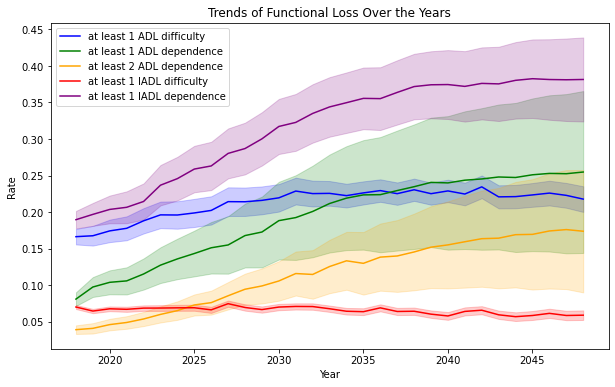


Fig S9. Probabilistic sensitivity analysis results of projections. Shaded areas represent the standard deviations of predictions in each year.

Table S1. The predictors in the logistic regression equations of two-year event probabilities.

|  | Memory  issue | Hypertension | diabetes | cancer | lung disease | heart problems | stroke | psychiatric diseases | arthritis | dyslipidemia | liver disease | kidney disease | digestive disorder | asthma |
| --- | --- | --- | --- | --- | --- | --- | --- | --- | --- | --- | --- | --- | --- | --- |
| Age splines | √ | √ | √ | √ | √ | √ | √ |  | √ |  |  |  |  | √ |
| BMI splines |  | √ | √ |  |  | √ |  |  |  | √ | √ |  | √ |  |
| Male |  | √ | √ | √ | √ | √ | √ |  | √ | √ | √ | √ |  |  |
| Rural |  | √ |  |  |  |  |  |  | √ | √ |  |  | √ |  |
| Married/partnered |  |  |  |  |  |  | √ |  |  |  |  |  |  |  |
| Education category |  | √ |  |  |  | √ |  |  | √ | √ | √ |  |  |  |
| Memory  issue |  |  | √ |  | √ | √ | √ | √ | √ |  |  |  |  |  |
| Hypertension |  |  | √ |  |  | √ | √ | √ |  | √ |  |  |  |  |
| Diabetes |  | √ |  |  |  | √ |  | √ |  | √ | √ |  |  |  |
| Cancer |  |  |  |  |  |  |  |  |  | √ |  |  |  |  |
| Lung disease |  |  | √ |  |  | √ | √ |  | √ |  | √ | √ | √ | √ |
| Heart disease | √ | √ |  |  | √ |  | √ |  |  | √ | √ | √ |  | √ |
| Stroke history | √ | √ |  |  |  |  |  | √ |  |  |  | √ |  |  |
| Psychiatric disease | √ |  | √ |  |  |  |  |  |  | √ | √ |  | √ |  |
| Arthritis |  | √ | √ | √ | √ |  |  | √ |  |  | √ | √ | √ |  |
| Dyslipidemia | √ | √ | √ | √ |  | √ | √ |  |  |  | √ | √ | √ | √ |
| Liver disease | √ |  |  | √ | √ |  |  |  |  | √ |  | √ | √ |  |
| Kidney disease |  |  | √ | √ | √ | √ |  | √ | √ |  | √ |  | √ |  |
| Digestive disease |  |  |  | √ | √ | √ |  | √ | √ | √ | √ | √ |  |  |
| Asthma |  |  |  | √ | √ | √ | √ | √ |  | √ |  |  |  |  |
| Drinking at least once a day |  | √ | √ |  |  | √ | √ | √ |  | √ |  |  |  |  |
| Smoking |  |  |  |  |  | √ |  |  | √ | √ |  |  |  | √ |
